# Analytical and Numerical Investigation of Nanowire Transistor X-ray Detector

**DOI:** 10.3390/ma16072637

**Published:** 2023-03-27

**Authors:** Abdelhady Ellakany, Abdelhalim Zekry, Mohamed Abouelatta, Ahmed Shaker, Gihan T. Sayah, Mohamed M. El-Banna

**Affiliations:** 1 Electronics and Communication Engineering Department, Faculty of Engineering, Ain Shams University, Cairo 11517, Egypt; 2 Engineering Physics and Mathematics Department, Faculty of Engineering, Ain Shams University, Cairo 11517, Egypt; 3 Electronic Engineering Department, Nuclear Material Authority, Cairo 11381, Egypt

**Keywords:** nanowire detectors, hard X-ray, InP, phototransistor, modeling

## Abstract

Recently, nanowire detectors have been attracting increasing interest thanks to their advantages of high resolution and gain. The potential of using nanowire detectors is investigated in this work by developing a physically based model for Indium Phosphide (InP) phototransistor as well as by performing TCAD simulations. The model is based on solving the basic semiconductor equations for bipolar transistors and considering the effects of charge distribution on the bulk and on the surface. The developed model also takes into consideration the impact of surface traps, which are induced by photogenerated carriers situated at the surface of the nanowire. Further, photogating phenomena and photodoping are also included. Moreover, displacement damage (DD) is also investigated; an issue arises when the detector is exposed to repeated doses. The presented analytical model can predict the current produced from the incident X-ray beam at various energies. The calculation of the gain of the presented nanowire carefully considers the different governing effects at several values of energies as well as biasing voltage and doping. The proposed model is built in MATLAB, and the validity check of the model results is achieved using SILVACO TCAD device simulation. Comparisons between the proposed model results and SILVACO TCAD device simulation are provided and show good agreement.

## 1. Introduction

Semiconductor nanowires (NWs) have demonstrated auspicious performance in multi-diverse fields such as light-emitting diodes [1], transistors [2,3,4], FETs [5], solar cells [6,7,8,9,10,11], lasers [12,13], and quantum physics [14], as well as their use in light detection in the infrared to the ultraviolet range [15,16,17,18,19,20,21,22]. Furthermore, X-ray detection has been exhibited in single NW detectors [23,24] thanks to their long penetration depth. The need for the enhanced spatial resolution of X-ray microscopy is increasing because of the growing attention to nanomaterials [25,26].

In recent experiments, hard X-ray beams was focused to less than 5 nm using Kirkpatrick–Baez (KB) mirrors and zone plates [27,28]. However, the spatial resolution of X-ray microscopy not only changes according to the spot size of the X-ray but also depends on the pixel size of the X-ray detector. As direct detection by semiconductor detectors is centered on the pixel size in multiples of 10 µm that have thicknesses of over 100 µm [29], there is a need for novel structures to cope with the spot size reduction. Moreover, there are main factors that are required to achieve high-resolution X-ray detection. These include a minimum active region and appropriate thickness suitable for radiation absorption. Based on the above key reasons, NW-shaped detectors have been suggested as appropriate X-ray detectors. A nanowire has optical features that show considerable size dependence, which renders them appealing contenders as light absorbers with a potential high gain. Thus, developing and understanding NW detectors is mandatory to meet future applications in distinctive fields [23].

Further, the main X-ray absorption scales in accordance with the volume, which instinctively indicates a low carrier concentration for small pixel sizes. Meanwhile, for NW structures, the lifetime of the secondary electron-hole pairs can be significantly different from bulk as the recombination can be influenced by surface states [23]. Furthermore, mobility reveals a decline at low doping levels [6]. Moreover, the high surface-to-volume ratio, as well as the occurrence of deep-level surface trap states in nanowires, extend the photo carrier lifetime, while decreasing the active area reduces the carrier transit time [16].

Direct detectors involve high-quality semiconductor materials that have low trap state densities to collect the photo-excited carriers before recombination due to the Shockley–Read–Hall (SRH) process. In this respect, materials such as Si and CdTe could be utilized for direct X-ray detection [30]. In addition, InP and InGaP nanowires have been utilized to develop X-ray detectors [26]. In [23], a 100 nm InP NW structure was employed to image an X-ray nano focus. Regarding this fabricated photoconductor structure, the induced conductance due to the incident X-ray dose was more than other reported results by five orders of magnitude. So, because of its high density, large atomic number, and relatively high intrinsic resistivity, InP material is a strong potential candidate for use in solid-state nuclear detectors. InP is supposed to provide superior quality as a solid-state detector [31].

Nanowire phototransistors can be a promising candidate for X-ray detection due to their unique properties and advantages over other X-ray detectors, such as photodiodes and photoconductors. A nanowire transistor has better detection sensitivity and higher image quality [32,33], in addition to its a relatively fast response time. Moreover, nanowire phototransistors can be easily integrated into existing microelectronic devices due to their small size and compatibility with standard microfabrication techniques. This reduces the cost and complexity of device manufacturing [34].

In this article, we present a proposed InP NW X-ray phototransistor detector. To provide design guidelines and to understand the operation of this novel structure, we introduce a physically based model that incorporates the impact of trap charges and other physical effects that are crucial in modeling NW structures. The model can predict the photoconductive current and gain in semiconductor NWs. The advantages of this model appear in the early design phase to develop a new NW detector. The proposed model results reveal the uniqueness of NWs for hard X-ray applications. In addition, the proposed model explains the charge trapping at surface states, which is known as the photogating and photodoping phenomenon. Furthermore, SILVACO TCAD device simulator is used to validate our proposed model and to further investigate some phenomena that cannot be predicted by the analytical model, such as the effect of biasing voltage on the behavior of the NW phototransistor and leakage current.

This article is planned as follows. In the following section, the main structure and design parameters are presented. In Section 3, the model description and equations are derived. In Section 4, the simulation techniques are presented and discussed. The results and discussions are provided in Section 5**,** including the NW device orientation along the X-ray beam to enhance the absorption and the investigation of the displacement damage effect on the behavior of the NW detector. Section 7 is dedicated to the conclusion of this work.

## 2. Main Structure and Design Parameters

As stated herein, there are some factors to drive the development of InP to be used in solid-state nuclear detectors. These factors include the main properties of InP, such as its density, atomic number, and energy gap. For InP, a high density (*ρ*) of approximately 4.79 g/cm^3^ and a large atomic number (*A* = 80.2) are reported in the literature. Furthermore, the bandgap energy is approximately 1.34 eV, which is considerably wide, resulting in allowing room temperature operation [31]. Therefore, InP is selected in our work as a favorable material for X-ray detection, to be investigated through analytical modeling and TCAD simulations.

**Figure 1:**
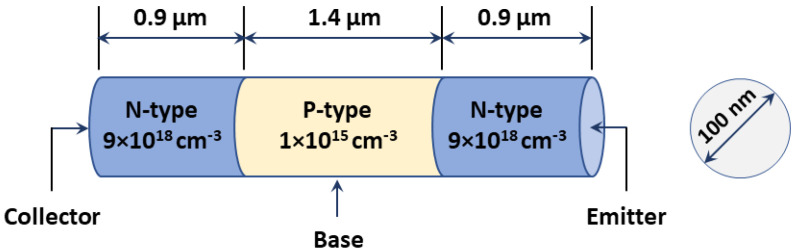
Schematic representation of the 3D NW detector used in our analysis.

The basic NW structure, presented here, is shown in Figure 1, and a list of the main design parameters is displayed in Table 1. It is similar to a previously fabricated device [35]; however, for the fabricated detector, it was a photoconductor in which only n-type doping is invoked, while, in our structure, we propose an NPN transistor. Our device is composed of three different regions, emitter, collector, and base, which is considered the active region. The distance between the two metal electrodes is 3.2 μm. The lengths of the different regions are 0.9, 1.4, and 0.9 µm for the collector, base, and emitter, respectively, while the NW diameter is 100 nm. An n-type region with a doping density of 9 × 10^18^ cm^−3^ (which can be obtained by the doping of S, Fe, or Sn) for the collector and emitter is used, while the middle base segment is doped with p-type with a doping density of 1 × 10^15^ cm^−3^ (which can be obtained by the doping of Zn). These values are similar to those used in [35], except for the doping type of the base, which is p-type. Regarding InP physical parameters, the mobility is taken as 200 cm^2^/V.s, and the recombination bulk lifetime is *τ_BG_* ≈ 1 ns [5,36]. The principal physical properties of a InP nanowire detector are listed in Table 2. 

## 3. Model Description

Notably, a conventional phototransistor is different from a bipolar transistor because it has a larger base-collector junction as a light absorber region [37,38]. On the other hand, in NW, the base-collector junction cross-section is the same as the base-emitter cross-section. Because the surface-to-volume ratio is large, a photogating process occurs. In this process, the trapped charges develop a virtual electrostatic gate, which alters the Fermi level and increases the conductance. Additionally, a photodoping process may occur in which holes are confined in long-lived traps. According to this phenomenon, additional conduction band electrons are produced, resulting in a possible conductivity rise [5,26].

**Figure 2:**
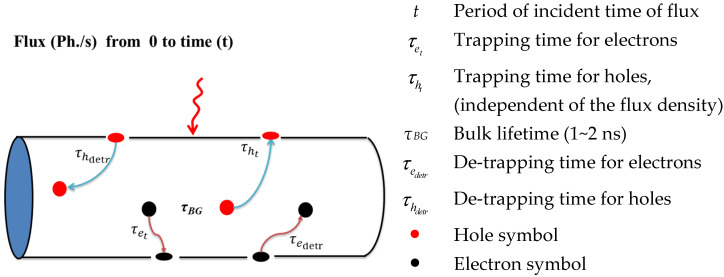
Representative diagram of the important terms of the NW phototransistor modeling.

Here, we present a mathematical derivation for the proposed model, starting from the basic semiconductor principles, to develop the photocurrent, gain, and signal-to-noise ratio. The principle physical terms used in the derivation are indicated in Figure 2. It should be pointed out here that the trapping (or de-trapping) time of electrons and holes is different; however, in our analysis, the minority carriers inside the base are of the most interest (electrons here). Therefore, our concern will be focused on the electron flow through the device structure. When the InP NW phototransistor is exposed to hard X-rays, the incident beam generates an electron-hole pair. The surface trap captures charges for a period equal to the trapping time (*τ_t_*), then the charges release from the surface for a period equal to the de-trapping time (*τ_detr_*), as displayed in Figure 2.

In our model, it is assumed that the electrons and holes generated in the photogeneration process will be directly trapped, assuming that the trapped electron density ($N_{t}^{-}\left(t\right)$) is changing with time, *t*. The traps will release electrons at a rate of $N_{t}^{-}(t)/\tau_{t}$, where *τ_t_* is the average time of traps (trapping time). This can be written as a rate equation,

(1) $$\frac{\partial N_{t}^{-}(t)}{\partial t}=\frac{-N_{t}^{-}(t)}{\tau_{t}}$$

where $N_{t}^{-}(t)$ is the trapping charge as a function of time, which is accumulated on the surface during the whole time of the incident beam (*t*). From Equation (1), one can easily obtain $N_{t}^{-}(t)$ as,

(2) $$N^{-}(t)=N_{tt}e^{-t/\tau_{t}}$$

where *N_tt_* is the total trap density, which is the number of trap charges per cm^3^. The trap charge on the surface, N(t), is equal to the total trapped charge, *N_tt_*, subtracted from the release charge, which is given by Equation (2). Therefore, the trapped charge on the surface is given as,

(3) $$N(t)=N_{tt}-N_{tt}e^{-t/\tau_{t}}=N_{tt}(1-e^{-t/\tau_{t}})$$

To obtain the dynamic response of the nanowire detector, the generation rate is calculated as a function of the flux density and the thickness. We assume that the incident flux is *φ_o_* (Ph./cm^2^/s). Then, the photon flux, upon traversing into the NW material, as a function of distance x is given by,

(4) $$\varphi (x)=\varphi_{o}e^{-\alpha x}$$

where *α* is the absorption coefficient. The absorbed photon flux in a thickness, *d*, is given by $\varphi_{o}(1-e^{-\alpha d})$. Assuming *αd* << 1 results in an absorbed photon flux of approximately $\varphi_{o}\alpha d$ [39,40,41]. Then, the number of generated electron-hole pairs is $\eta \varphi_{o}\alpha d$, where η is the number of the created electron-hole pairs per X-ray photon, which is equal to 3290 electron-hole pairs at 13.8 keV photon energy [14]. Thus, the generation rate, *g* (per unit volume), can be expressed by,

(5) $$g=\frac{\eta \varphi_{o}\alpha d}{d}=\eta \alpha \varphi_{o}$$

This is correct when the time is insufficient to deplete all the trapped electrons. After the incident beam is switched ON, the corresponding boundary condition is $N^{-}$(0) = 0. The overall dynamic response is expressed by the rate equation [42],

(6) $$\frac{\partial N^{-}(t)}{\partial t}=g-\frac{N^{-}(t)}{\tau_{t}}$$

The solution of the previous equation, based on the appropriate initial condition, is,

(7) $$N^{-}(t)=g\tau_{t}\left(1-e^{-t/\tau_{t}}\right)$$

The steady state trap density after a significant amount of time is *gτ_t_*, as verified in [43]. Similarly, the reduction of the traps is exponential according to the following equation when the beam is switched OFF,

(8) $$N^{+}(t)=N^{+}(0)e^{-t/\tau }{}^{{}_{\mathrm{detr}}}$$

where $N^{+}(0)$ is the full trap charge at the X-ray beam switched OFF, and *τ_detr_* is the de-trapping time. The de-trapping electrons will be free within the base, and their rate will constitute the base current, which is provided by [43],

(9) $$I_{B}=\frac{qAN^{-}(t)W_{B}}{\tau_{t}}$$

where *W_B_* is the base width and *A* is the NW cross-sectional area. Additionally, the collector current (*I_C_*) will be formed by the diffusion of this charge towards the collector junction, as given by [43],

(10) $$I_{C}=\frac{qAN^{-}(t)W_{B}}{T_{c}}p_{trap}$$

where *p_trap_* is the trapping probability and *T_c_* is the transient time towards the collector, which is given by [37],

(11) $$T_{C}=\frac{W_{B}^{2}}{2D_{n}}$$

where *D_n_* is the diffusion constant, which is related to the mobility by the well-known Einstein relation. Notably, the transit time of the minority carriers in the base puts an upper limit for the effectiveness of a transistor to operate as an amplifier [20]. Then, the gain (*β*) is given by,

(12) $$\beta =\frac{I_{C}}{I_{B}}=\frac{\tau_{t}}{T_{C}}p_{trap}$$

Because the lifetime is *τ_t_* = 0.3 s at 2 × 10^9^ Ph./s, the lifetime is inversely proportional to the flux according to *τ_t_* ≈ 6.0 × 10^8^/Φ [23]. Thus, *T_c_* can be calculated as,

$$T_{c}=\frac{{\left(1.4\times 10^{-4}\right)}^{2}}{2\times 200\times 0.025}=1.895\;\mathrm{ns}$$

For *p_trap_* ≈ 2.3 × 10^−6^, the gain can be estimated as *β* = 0.36 × 10^3^, 2.0802 × 10^3^, and 1.2135 × 10^4^ for fluxes of 2.0 × 10^9^, 3.5 × 10^8^, and 6.2 × 10^7^ (Ph./s), respectively. Although the lifetimes are much higher than *τ_BG_*, the gain is relatively low because only a fraction, *p_trap_*, of the generated holes is trapped. Thus, the main factor of the gain decline is according to the low trapping probability. However, the gain of the transistor with the trapping charge is still higher than that expected from a scaling of bulk parameters [23].

The relation between the collector current and the base current is provided by the following equation [37],

(13) $$I_{C}=(I_{B}+I_{CO})(\beta +1)$$

where *I_C_* is the collector current, *I_B_* is the base current, *I_CO_* is the leakage current, and finally, *β* is the gain of the phototransistor detector; the above equation is valid only when the base is open.

Next, the signal-to-noise ratio is given by the following equation [38],

(14) $$\frac{S}{N}=\frac{i_{p}^{2}}{<i_{s}^{2}>+<i_{T}^{2}>}=\frac{\left(1/2\right){\left(q\eta p_{opt}/h\upsilon \right)}^{2}}{2qI_{eq}B+4kTB/R_{eq}}$$

where *B* is the bandwidth, assumed to be 1 Hz, *η* is the number of the electron-hole generated per photon and equals 3290. The thermal noise is given by the following equation [38,44],

(15) $$i_{T}^{2}=4kTB/R_{eq}$$

*I_eq_* is the equivalent noise current and is given by [41],

(16) $$I_{eq}=I_{CEO}\left(1+\frac{2h_{fe}{}^{2}}{h_{FE}}\right)$$

where *h_fe_* is the incremental common-emitter current gain. In addition, the noise-equivalent power (NEP) is calculated to study the noise at different beam energies, and the NEP is given by the following expression (when S/N = 1, *B* = 1 Hz) [38],

(17) $$NEP=\left(\frac{h\upsilon }{\eta }\right)\sqrt{\frac{2I_{eq}}{q}}$$

The NEP can be calculated as 1.0325 × 10^4^, 4.2754 × 10^3^, and 1.7796 × 10^3^ keV at 2 × 10^9^, 3.5 × 10^8^, and 6.2 × 10^7^ Ph./s, respectively. The incremental common-emitter current gain (*h_fe_*) is given by the following equation [44],

(18) $$h_{fe}=\frac{dI_{C}}{dI_{B}}$$

At the same value of *V_ce_*, the magnitude of *h_fe_* and *h_FE_* will be the same at every point on the characteristics if *I_CEO_* = 0 µA, or very small [44]. The calculated values of gain, NEP and S/N are listed as functions of the photon flux as illustrated in Table 3.

## 4. TCAD Simulation Techniques

**Figure 3:**
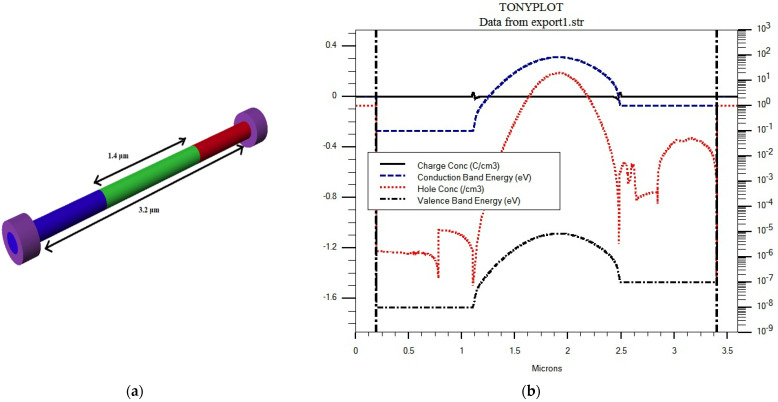
(**a**) 3D the nano wire detector and (**b**) Energy-band diagram under bias for a simulation case study.

Regarding the simulation of the presented structure in the TCAD environment, we began with the DevEdit module within SILVACO TCAD to generate the structure by defining the regions, materials, doping densities, and electrodes of the structure, and then an appropriate mesh is created. A 3D view of the generated structure is displayed in Figure 3a. To test the structure operation, we used the Atlas module [45] to simulate the structure. In the device simulator Atlas, the fundamental differential equations of semiconductor physics are concurrently solved with appropriate boundary and initial conditions. As a first test, the InP NW phototransistor is subjected to nano-focused hard X-rays, and the corresponding energy band diagram is demonstrated in Figure 3b. As shown in the figure, the accumulation of holes increases the potential and, in turn, permits a larger flow of electrons from the emitter region to the collector region.

### 4.1. Simulation Models

In our simulation, the bipolar models are applied, which include a general mobility model that enables concentration and temperature dependency. In addition, the recombination models are enabled, such as Concentration Dependent SRH (CONSRH) and auger model (AUGER) [45]. In the concentration Dependent SRH (CONSRH) recombination mode, the lifetimes are taken to be dependent on concentration. Regarding the Auger recombination model, a dependence of recombination lifetime on carrier density is utilized, which is significant at quite high carrier densities [45]. Further, carrier Fermi statistics and Bandgap Narrowing (BGN) models are included [45].

In addition, the capability of a single event upset/photogeneration transient simulation is included in 2D and 3D using the SINGLE-EVENT-UPSET (SEU) statement. It allows specifying the radial, length, and time dependence of generated charge along the tracks. There can be a single particle strike or multiple strikes that can be inputted by the user [45]. Additionally, a user-defined SEU can be defined by the user by incorporating C-Interpreter to generate any arbitrary generation rate profile as a function of time and/or position. The parameters of the SEU statement, such as start time, end time, and step time, are defined in the solve statement. In our simulation, start, end, and step times are chosen to be 0, 4.9, and 10^−4^ s, respectively. The X-ray beam can be incident in two ways, either vertical (parallel to the NW) or horizontal (perpendicular to the NW), and the two cases are explained in detail and compared to each other.

The maximum charge of the incident beam equals *e × N* [46], where *e* is the charge of electron and *N* is the number of electrons and holes generated by incident beam energy. The electron-hole pair generated within this process is *η* = *E*/𝜖, where *E* is the incident X-ray beam energy, and *𝜖* is the ionization energy. In addition, the charge generation rate can be computed by specifying the bandgap energy by the analytical equation 𝜖 = 2.75*E*_g_ + 0.55 eV [47]. The generation rate (in cm^−3^s^−1^) in terms of the photon flux, ϕ, can be expressed by the following equation,

(19) $$G_{n,p}=\frac{\eta P_{abs}\phi }{Ad}$$

where *P_abs_* is the X-ray absorption probability. The area of the incident X-ray beam is denoted by *A*, *d* is the thickness of the InP material, and *η* is the number of electron-hole pairs generated by every photon of the X-ray beam. The maximum absorption probability is *P_abs_* = 6.2 × 10^−4^. It is well-known that a single X-ray absorption event produces 3290 conduction band electron-hole pairs at 13.8 keV photon energy [47].

### 4.2. Simulation Tools Calibration

**Figure 4:**
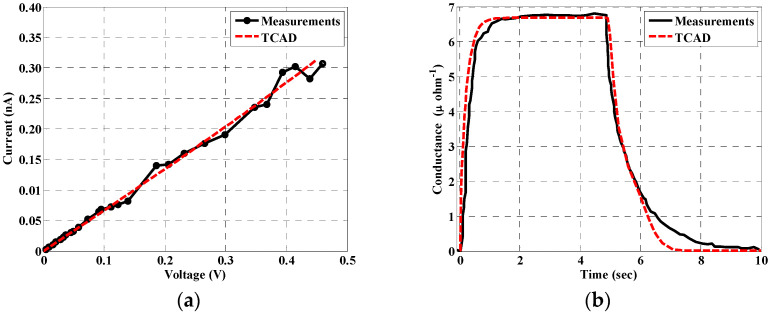
(**a**) Leakage Current of the NW photoconductor. (**b**) Current of the NW photoconductor detector as a function of time at values of flux is 2 × 10^9^ (Ph./s). Measurements are extracted from REF [23].

Calibration is one of the major concerns encountered by TCAD device simulation. SILVACO TCAD tools incorporate advanced and accurate physical models for a huge variety of devices. However, in order to check the validity and reliability of the simulation when new devices or materials are concerned, one has to calibrate the models and parameters versus experimental work. Here, we calibrate our simulation model, including the physical models and material parameters versus the published measurements of an InP-based X-ray detector. The parameters, incorporated into the simulation, are surface trap level, bandgap, mobility of holes and electrons, and other physical model parameters. In addition, the lifetime is taken as a function of flux density according to REF [23]. Two different conditions are considered. The first simulation is performed when the device is not exposed to the X-ray beam. In this context, the leakage current is displayed in Figure 4a. The second simulation is performed when the device is exposed to the X-ray beam at 2 × 10^9^ (Ph./s). The conductance as a function of time is illustrated in Figure 4b when the X-ray beam is turned ON at 0 s and turned OFF at 4.9 s. For both cases, the simulation results are compared with measurements for a single 100 nm diameter InP nanowire [23].

## 5. Results and Discussions

The physics-based model formulated herein is crucial for determining the dependence of the device performance on various design parameters, such as doping concentration, diameter, and surface charges. A critical balance between these design parameters is needed to maximize the photo transitive gain and minimize the leakage current. Therefore, the effect of both radiation flux and doping concentration on the gain is studied. The impact of both radiation flux and biasing voltage on the leakage current is also investigated.

### 5.1. Leakage Current

**Figure 5:**
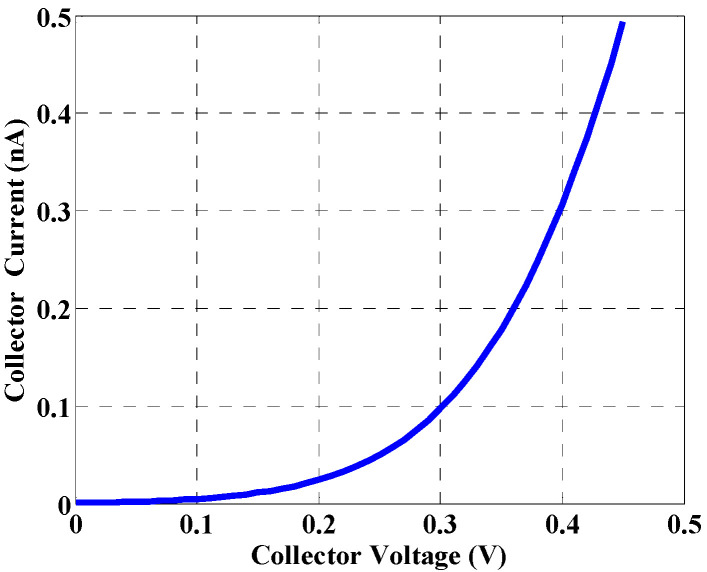
Leakage current of the NW phototransistor detector.

Remarkably, the dark current is a key factor, which is used as a measure of effective operation of the detectors. Minimal leakage current is a crucial factor that defines a detector quality [37]. In addition, the leakage current determines the minimum detectable signal strength. The leakage current is calculated based on the TCAD simulation, and it is found to be approximately 0.49 nA at 0.45 V, as shown in Figure 5. Unfortunately, the results show that the leakage current is amplified by the same gain factor as given in Equation (13).

### 5.2. Transient Simulation

**Figure 6:**
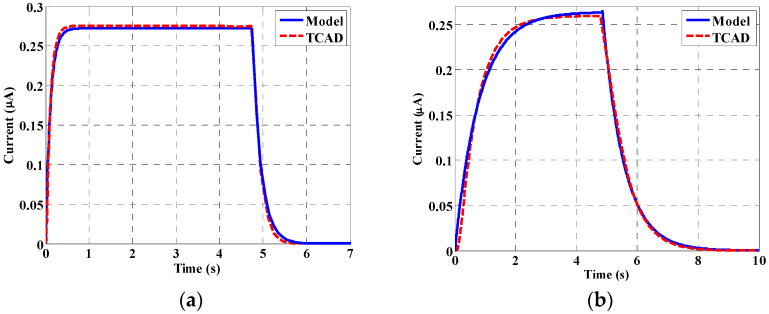

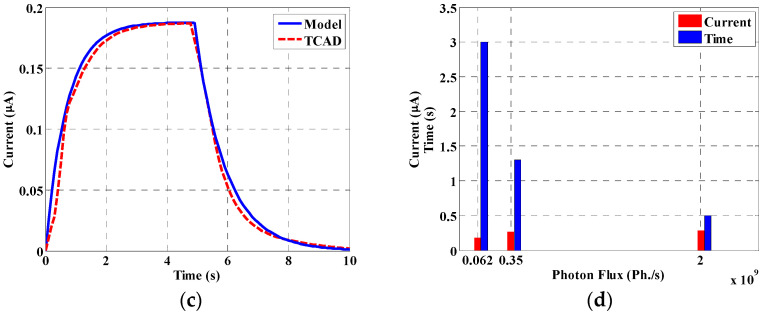
Current of the NW detector as a function of time for various values of flux, (**a**) 2 × 10^9^ Ph./s.; (**b**) 3.5 × 10^8^ Ph./s.; (**c**) 6.2 × 10^7^ Ph./s.; (**d**) rise time and maximum current variation with flux.

When the proposed device is subjected to X-ray beams, the electric current changes from 0.49 nA (corresponding to dark condition) to 0.28 µA, 0.265 µA, and 0.18 µA at a flux of 2 × 10^9^, 3.5 × 10^8^, and 6.2 × 10^7^ Ph./s., as shown in Figure 6a, Figure 6b, and Figure 6c, respectively. In these simulations, the incident beam was turned ON at *t* = 0 s, while it was turned OFF at *t* = 4.9 s. The rise time and maximum current are also shown in Figure 6d for the different values of the flux. The rise time is calculated as the time at which the current reaches 90% of its maximum. Both the TCAD simulation and model results are shown, revealing a good agreement. As can be depicted in the figure, collection time is significant, which is a main disadvantage of the NW detector. These results could be explained by photogating and photo doping impacts, which have been reported in experimental studies on NW detectors based on X-ray and UV excitation [21].

### 5.3. Effect of Doping Concentration on the Gain

**Figure 7:**
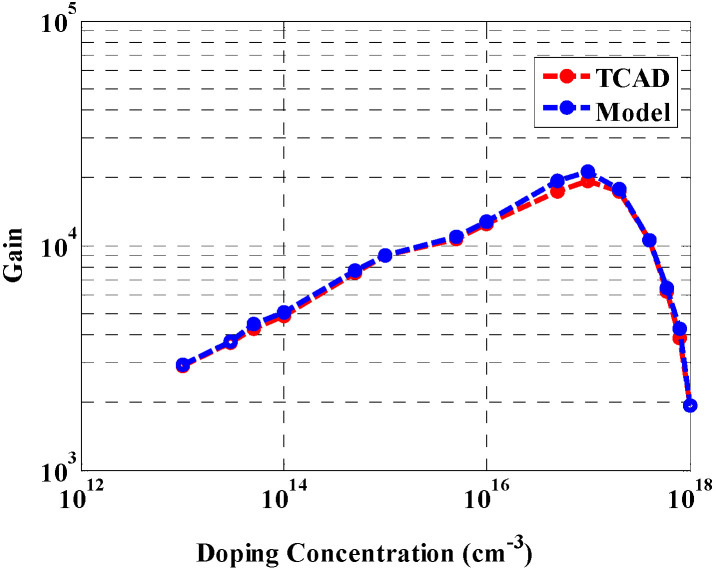
NW gain vs. doping concentration.

At low doping concentrations, the surface states can be fully depleted, but the gain is low due to the low impurities within the NW, which are trapped on the surface. Thus, the photogating phenomena impact is minor. As doping increases, on the other hand, more holes are trapped at the surface, which leads to a rise in the surface charge, which in turn increases the gain, as shown in Figure 7. At a certain limit, the gain shows a decline, as demonstrated in the figure, as the surface states become unable to trap additional ionized holes [43]. It should be pointed out that mobility degradation due to high doping is taken into consideration.

### 5.4. Effect of Flux Density on the Transistor Gain

**Figure 8:**
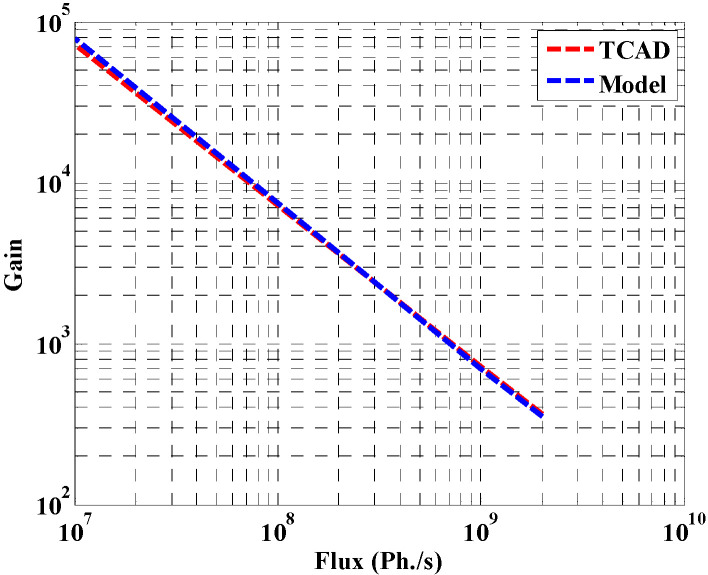
Relation between gain and flux density (Ph./s).

The dependence of gain on flux density is also investigated through the TCAD simulation and our model, as shown in Figure 8. At low flux intensity, the gain of the proposed structure is high because of the low charge density at the surface. In addition, at low flux, the lifetime exceeds 1 min, while it is about 0.3 s at full flux [23]. At high flux values, the lifetime is inversely proportional to the flux, which means that the de-trapping rate is also proportional to the flux [23]. Increasing the photo generation rate has the effect of drastically reducing the carrier lifetimes, which lowers the gain, as evident in Figure 8.

### 5.5. Effect of Biasing Voltage on the Transistor Current

**Figure 9:**
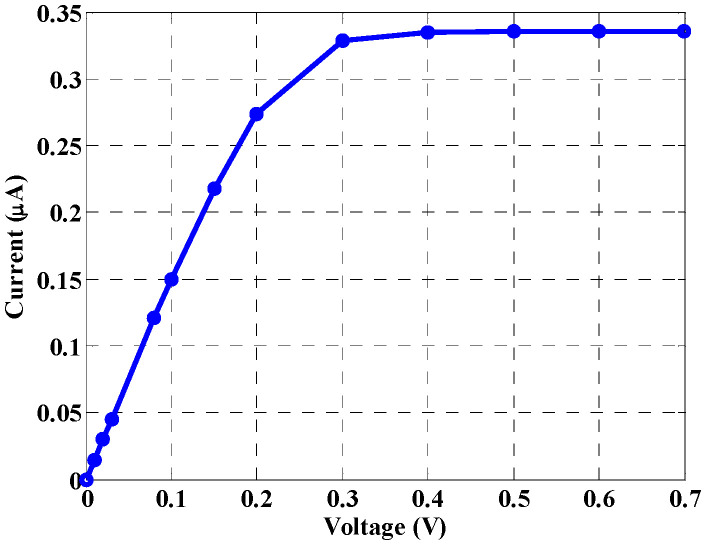
Relation between biasing voltage and output current.

Here, an X-ray beam with a flux up to 2 × 10^9^ ph./s is incident on the NW detector, and the current is calculated at different biasing voltages using SILVACO TCAD. The I-V characteristic is displayed in Figure 9. The figure demonstrates that when the biasing voltage increases, a linear increase in the current occurs until the biasing voltage reaches 0.3 V, and then the current almost saturates. Figure 9 shows that the suitable biasing voltage is less than 0.3 V because, if the biasing voltage is increased by more than the 0.3 V, the leakage current will increase without increasing the output current. The explanation of this trend is as follows. For low voltage values, the electric field is low, and the drift velocity is consequently proportional to the electric field. For higher voltages, the field is considerably high, and mobility becomes nonlinear, causing a saturation of drift velocity, also implying a saturation in the current, as indicated in Figure 9.

### 5.6. Effect of Flux Density on the Current

**Figure 10:**
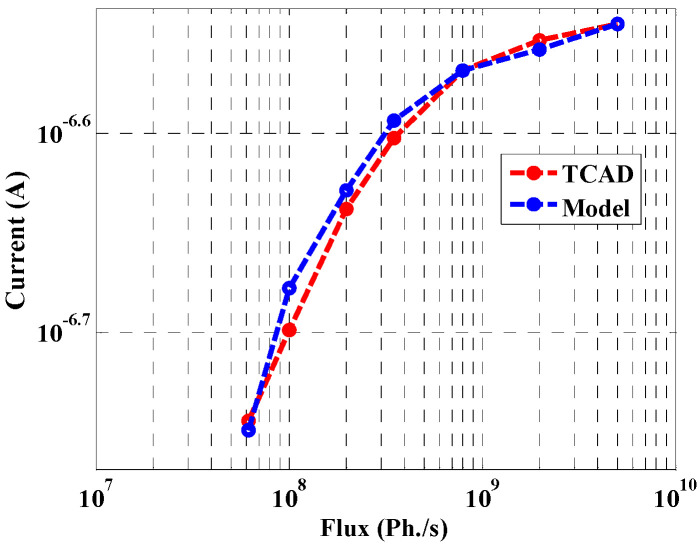
Maximum current vs. X-ray flux density (Ph./s).

The conductivity strongly increases by raising the X-ray flux. Thus, the current increases linearly with the increase of the flux. This phenomenon is illustrated in Figure 10, which shows the variation of the detector current versus flux density. The results are shown for both the TCAD simulation and our analytical model. The current increases as expected until the flux reaches a value of 5 × 10^8^ ph./s, and then the current nearly saturates as shown. The interpretation of this trend is that the lifetime decreases as the flux density increases, leading to an increase in the recombination rate. Thus, the mobility saturates, and the current becomes almost flat, as shown in Figure 10.

### 5.7. Effect of Orientation of the X-ray Beam

**Figure 11:**
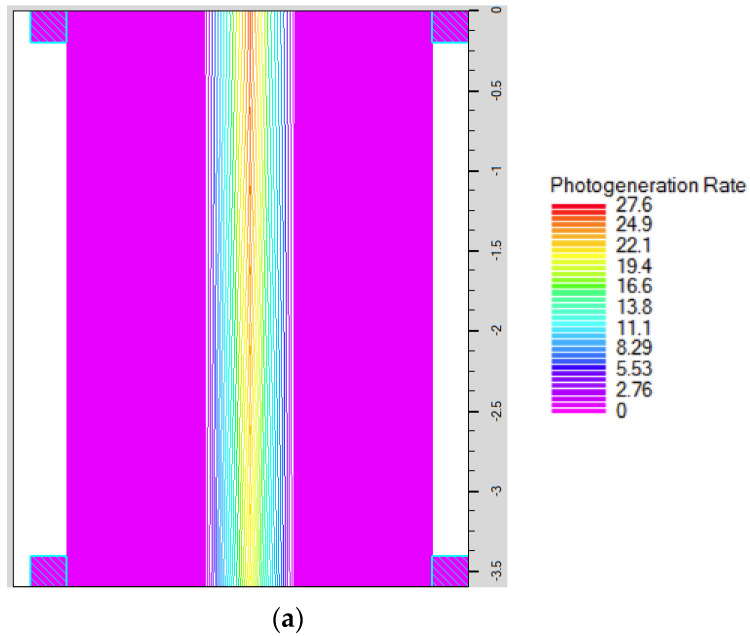

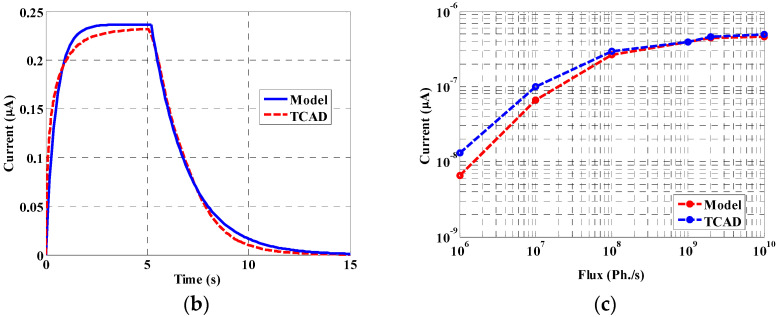
Nanowire structure oriented along X-ray beam: (**a**) Attenuation along the nano wire detector in the Z direction, (**b**) Current of the NW detector as a function of time for a flux of 2 × 10^9^ Ph./s, and (**c**) Maximum current versus flux density.

Notably, a light beam, upon its incidence on a material, will be absorbed near the surface if the absorption coefficient of the material is high. On the other hand, if the absorption coefficient is relatively low, the light beam can penetrate deeper inside the material [28]. Therefore, we will study a different orientation of our NW transistor along the X-ray beam in order to investigate the possibility of more efficient energy absorption. Here, the incident beam is assumed to be parallel to the NW axis, and it is absorbed efficiently because the thickness of the NW detector is high, so the detector gives high probability to absorb the beam energy along the axis of the NW device, as shown in Figure 11a. As can be inferred from the figure, the photogeneration rate is maximum at the top in the center of the nanowire (the generation rate equals 27.6 (1/cm^3^)). Then, the charge is trapped at the surface and the photogeneration rate decreases along the nanowire direction according to the attenuation coefficient of the InP material (the generation rate equals 19.4 (1/cm^3^)). For this configuration, the X-ray absorption can happen along the NW length, while the spatial resolution is limited by the diameter of the NW device [48]. The current of the NW detector as a function of time for a flux of 2 × 10^9^ Ph./s is displayed in Figure 11b, while Figure 11c reports the maximum current versus flux density. It is noted that the simulation conditions, performed in this part, were the same as those applied in Section 5.3 for both the analytical model and the TCAD simulations.

It can be inferred from the figure that, in this case of parallel beam incidence to the NW axis, the output current is less than that in Figure 6a, although the incident beam energy is the same value (2 × 10^9^ Ph./s). Thus, the gain, in this case, is less than that obtained previously for perpendicular absorption due to the low trapping probability at the base region and the encountered long distance, which makes the carriers take more time, thus the probability of the recombination process is increased.

## 6. Effect of Displacement Damage

**Figure 12:**
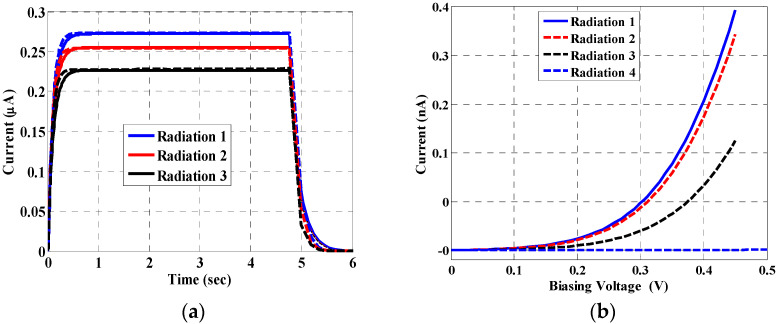
(**a**) Output current due to displacement damage. Lines: TCAD and dotted: Model, and (**b**) Leakage current due to displacement damage.

When energetic particles collide with the semiconductor lattice, the atoms may become dislocated from their lattice site and pushed into interstitial positions within the crystal. This cascade of collisions caused by energetic particle irradiation can result in a large, disordered region called a defect cluster [49]. Displacement damage is a complex function of radiation type, radiation energy, flux/dose rate, and pulsed or continuous irradiation. Vacancies are mobile at room temperature and can combine with donor atoms. The general result from energetic particle irradiation is the production of defects within the bandgap. Thus, this process changes the behavior of the detectors. Although there is no reported data in the literature on displacement damage from X-ray to bulk InP, some research studies revealed this effect on nanowires fabricated from different materials [50,51,52]. Therefore, in this section, we theoretically investigate the effect of displacement damage that may occur in InP nanowires to gain more insight towards the development of this type of detector. Figure 12 demonstrates that as the X-ray beam is incident multiple times on the InP nanowire, both output and leakage currents decrease due to displacement damage defects.

In our simulation and modeling, this effect of displacement damage is accomplished by using the radiation fluence model, which allows the simulation of the defect dislocation generation rate due to energetic particle bombardment in the semiconductor. The results are shown in Figure 12, demonstrating the current for a value of the radiation flux of 2 × 10^9^ Ph./s. This incidence is repeated three consecutive times. These results show that the detector gives less current upon increasing the radiation, as expected. In addition, the impact of the displacement damage on the leakage current is explored, as shown in Figure 12. When the NW phototransistor is exposed to radiation, the leakage current is decreased because traps due to displacement damage impact the density of space charge in bulk, as well as the recombination statistics. The provided results indicate the importance of including such an effect, which should be taken into consideration when designing an InP-based NW detector.

Finally, a comparison between different NW detector materials is presented to highlight the state-of-the-art, as indicated in Table 4. Ultra-high resolution X-ray detection in a single NW device, comprised of InGaP with a diameter of 175 nm, has been demonstrated, but the detector has a low gain [26]. On the other hand, a 100 nm NW, based on an FET structure, from InP has a high gain [23]. In addition, the fabrication of a phototransistor based on ZnO nanowire has been reported [16], which gives the highest gain but with the widest NW diameter. Additionally, the silicon NW phototransistor response was investigated under the influence of the visible wavelength region and ultraviolet, reporting a gain of 10^3^~10^5^ [43]. Our work demonstrates a proof-of-concept of a novel design of a NW InP, which is based on a phototransistor and gives a comparable gain. More investigations are needed to provide enhancement of our proposed design in order to bring such technology closer to the fabrication phase.

## 7. Conclusions

In this work, a simple analytical model based on solving the basic semiconductor equations for bipolar transistors has been presented to describe the behavior of a proposed NW phototransistor detector, which is based on InP material. The model takes into consideration the impact of charge distribution in materials, especially the trap charges on the surface of the nanowire detectors. The proposed model is built in MATLAB and compared to a simulated model by applying SILVACO TCAD device simulation to check its applicability. In addition, the comparison between the proposed model results and the SILVACO TCAD simulator results shows good agreement that validates the presented model.

The effects of various parameters on the device behavior were carefully studied and illustrated. The leakage current of the designed detector was found to be approximately 0.49 nA at 0.45 V. Regarding the transient simulation, the electric current changes from 0.49 nA (corresponding to dark condition) to 0.28 µA, 0.265 µA, and 0.18 µA at a flux of 2 × 10^9^, 3.5 × 10^8^, and 6.2 × 10^7^ Ph./s., respectively. The influence of doping concentration on the gain has also been studied. A deterioration in the gain was observed beyond doping of approximately 1 × 10^17^ cm^−3^, which is due to mobility degradation. Further, the impact of flux density on the transistor gain, and on the detector current, was explored. Moreover, the effect of biasing voltage on the transistor current was examined. According to this study, an appropriate design of the operating voltage of less than 0.3 V is recommended. We also studied the detector performance upon modifying the orientation of the X-ray beam. The effect of displacement damage was finally investigated, demonstrating the reduction of the detector current when repeating the X-ray incidence three consecutive times. Based on the developed analytical and TCAD simulation performed in this work, the proposed structure can be a potential candidate for the design of future X-ray NW phototransistor detectors.

## Figures and Tables

**Table 1:** Principal technological parameters of the proposed nanowire detector [5,36].

Description	Value	Unit
Nanowire diameter	100	nm
N-doping concentration	10^19^	cm^−3^
P-dope concentration	10^15^	cm^−3^
Active region length	1.4	µm
Length between electrodes	3.2	µm

**Table 2:** Physical parameters of the InP material [5,36].

Description	Value	Unit
Bulk Lifetime (*τ_BG_*)	10^−9^	s
Mobility (*µ*)	200	cm^2^/V.s
Energy gap (*E_eg_*)	1.34	eV
Density (*ρ*)	4.79	g/cm^3^
Atomic number: Indium (In): Phosphorus (P)	49 16	

**Table 3:** Gain, NEP, and S/N as functions of the flux density.

Photon Flux (Ph./s)	Gain	NEP (keV)	S/N
6.2 × 10^7^	1.214 × 10^4^	1.0325 × 10^4^	70.71
3.5 × 10^8^	2.080 × 10^3^	4.2754 × 10^3^	79.07
2.0 × 10^9^	0.360 × 10^3^	1.7796 × 10^3^	83.72

**Table 4:** Figures-of-merit in typical low-dimensional photodetectors.

Structure	Material	Diameter (nm)	Gain	Detection Range	REF
Phototransistor (NPN)	InP	100	0.36 × 10^3^~10^4^	X-ray	Our work
FET (N^+^NN^+^)	InP	100	10^3^~10^5^	X-ray	[23]
FET (N^+^NN^+^)	InP	60	0.34	X-ray	[25]
Phototransistor (NPN)	Si	100	10^3^~10^5^	UV	[43]
FET (N^+^NN^+^)	InGaP	175	0.34	X-ray	[26]
Phototransistor (NPN)	InP	53.2		VL	[53]
FET (N^+^NN^+^)	ZnO	1200	2 × 10^8^	UV	[16]

## Data Availability

Not applicable.

## References

[1] Kivisaari P., Berg A., Karimi M., Storm K., Limpert S. (2017). Optimization of Current Injection in AlGaInP Core-Shell Nanowire Light-Emitting Diodes. Nano Lett..

[2] Wernersson L.E., Thelander C., Lind E., Samuelson L. (2010). III–V Nanowires-extending a narrowing road. Proc. IEEE.

[3] Kim D.M., Jeong Y.H. (2014). Nanowire Field Effect Transistors: Principles and Applications.

[4] Yang B., Buddharaju K.D., Teo S.H.G., Singh N., Lo G.Q. (2008). Vertical Silicon-Nanowire Formation and Gate-All-Around MOSFET. IEEE Electron Device Lett..

[5] Wallentin J., Ek M., Wallenberg L.R., Samuelson L., Borgström M.T. (2012). Electron Trapping in InP Nanowire FETs with Stacking Faults. Nano Lett..

[6] Garnett E.C., Brongersma M.L., Cui Y., McGehee M.D. (2011). Nanowire solar cells. Annu. Rev. Mater. Res..

[7] Wallentin J., Anttu N., Asoli D., Huffman M., Åberg I., Magnusson M.H., Siefer G., Fuss-Kailuweit P., Dimroth F., Witzigmann B. (2013). InP nanowire array solar cells achieving 13.8% efficiency by exceeding the ray optics limit. Science.

[8] Prashant D.V., Agnihotri S.K., Samajdar D.P. (2022). Efficient GaAs nanowire solar cells with carrier selective contacts: FDTD and device analysis. Mater. Sci. Semicond. Process..

[9] Im J.H., Luo J., Franckevičius M., Pellet N., Gao P., Moehl T., Zakeeruddin S.M., Nazeeruddin M.K., Grätzel M., Park N.G. (2015). Nanowire perovskite solar cell. Nano Lett..

[10] Patzke G.R., Kontic R., Shiolashvili Z., Makhatadze N., Jishiashvili D. (2012). Hydrazine-assisted formation of indium phosphide (InP)-based nanowires and core-shell composites. Materials.

[11] Otnes G., Barrigón E., Sundvall C., Svensson K.E., Heurlin M. (2018). Understanding InP Nanowire Array Solar Cell Performance by Nanoprobe-Enabled Single Nanowire Measurements. Nano Lett..

[12] Maslov A.V., Ning C.Z. (2003). Reflection of guided modes in a semiconductor nanowire laser. Appl. Phys. Lett..

[13] Greytak A.B., Barrelet C.J., Li Y., Lieber C.M. (2005). Semiconductor nanowire laser and nanowire waveguide electrooptic modulators. Appl. Phys. Lett..

[14] Mourik V., Zuo K., Frolov S.M., Plissard S.R., Bakkers E.P.A.M., Kouwenhoven L.P. (2012). Signatures of Majorana fermions in hybrid superconductor-semiconductor nanowire devices. Science.

[15] Wang J.F., Gudiksen M.S., Duan X.F., Cui Y., Lieber C.M. (2001). Highly polarized photoluminescence and photodetection from single indium phosphide nanowires. Science.

[16] Soci C., Zhang A., Xiang B., Dayeh S.A., Aplin D.P.R., Park J., Bao X.Y., Lo Y.H., Wang D. (2007). ZnO nanowire UV photodetectors with high internal gain. Nano Lett..

[17] Pettersson H., Trägårdh J., Persson A.I., Landin L., Hessman D., Samuelson L. (2006). Infrared photodetectors in heterostructure nanowires. Nano Lett..

[18] Pettersson H., Zubritskaya I., Nghia N.T., Wallentin J., Borgström M.T. (2012). Electrical and optical properties of InP nanowire ensemble p+−i−n+ photodetectors. Nanotechnology.

[19] Jain V., Nowzari A., Wallentin J., Borgström M.T., Messing M.E. (2014). Study of photocurrent generation in InP nanowire-based p+-i-n+ photodetectors. Nano Res..

[20] Favre-Nicolin V., Mastropietro F., Eymery J., Camacho D., Niquet Y.M., Borg B.M., Messing M.E., Wernersson L.E., Algra R.E., Bakkers E.P.A.M. (2010). Analysis of strain and stacking faults in single nanowires using Bragg coherent diffraction imaging. New J. Phys..

[21] Newton M.C., Leake S.J., Harder R., Robinson I.K. (2010). Threedimensional imaging of strain in a single ZnO nanorod. Nat. Mater..

[22] Diaz A., Mocuta C., Stangl J., Mandl B., David C., Vila- Comamala J., Chamard V., Metzger T.H., Bauer G. (2009). Coherent diffraction imaging of a single epitaxial InAs nanowire using a focused X-ray beam. Phys. Rev. B.

[23] Wallentin J., Osterhoff M., Wilke R.N., Persson K.-M., Wernersson L.-E., Sprung M., Salditt T. (2014). Hard X-ray Detection Using a Single 100 nm Diameter Nanowire. Nano Lett..

[24] Zapf M., Ritzer M., Liborius L., Johannes A., Hafermann M., Schonherr S., Segura-Ruiz J., Martinez-Criado G., Prost W., Ronning C. (2020). Hot electrons in a nanowire hard X-ray detector. Nat. Commun..

[25] Chayanun L., Hrachowina L., Björling A., Borgström M.T., Wallentin J. (2020). Direct three-dimensional imaging of an X-ray nanofocus using a single 60 nm diameter nanowire device. Nano Lett..

[26] Chayanun L. (2020). Nanowire Devices for X-ray Detection. Ph.D. Thesis.

[27] Mimura H., Handa S., Kimura T., Yumoto H., Yamakawa D., Yokoyama H., Matsuyama S., Inagaki K., Yamamura K., Sano Y. (2010). Breaking the 10-nm barrier in hard-X-ray focusing. Nat. Phys..

[28] Döring F., Robisch A.L., Eberl C., Osterhoff M., Ruhlandt A., Liese T., Schlenkrich F., Hoffmann S., Bartels M., Salditt T. (2013). Sub-5 nm hard X-ray point focusing by a combined Kirkpatrick-Baez mirror and multilayer zone plate. Opt. Express.

[29] Ellakany A., Abouelatta M., Shaker A., Sayah G.T., El-Banna M. (2017). TCAD simulation of a proposed 3D CdZnTe detector. J. Eng..

[30] Kamehama H., Kawahito S., Shrestha S., Nakanishi S., Yasutomi K., Takeda A., Tsuru T.G., Arai Y. (2018). A low-noise X-ray astronomical silicon-on-insulator pixel detector using a pinned depleted diode structure. Sensors.

[31] Suzuki Y., Fukuda Y., Nagashima Y., Kan H. (1989). An indium phosphide solid state detector: A possible low energy gamma and neutrino detector. Nuclear Instruments and Methods in Physics Research Section A: Accelerators, Spectrometers. Detect. Assoc. Equip..

[32] Ko W.S., Bhattacharya I., Tran T.T.D., Ng K.W., Adair Gerke S., Chang-Hasnain C. (2016). Ultrahigh responsivity-bandwidth product in a compact InP nanopillar phototransistor directly grown on silicon. Sci. Rep..

[33] Ko W.S., Bhattacharya I., Tran T., Ng K.W., Chang-Hasnain C. InP nanowire avalanche photodiode and bipolar junction phototransistor integrated on silicon substrate. Proceedings of the 26th International Conference on Indium Phosphide and Related Materials (IPRM).

[34] Jeddi H. (2022). InP/InAsP Quantum Discs-in-Nanowire Array Photodetectors: Design, Fabrication and Optical Performance. Ph.D. Thesis.

[35] Minot E.D., Kelkensberg F., Van Kouwen M., Van Dam J.A., Kouwenhoven L.P., Zwiller V., Borgström M.T., Wunnicke O., Verheijen M.A., Bakkers E.P. (2007). Single quantum dot nanowire LEDs. Nano Lett..

[36] Joyce H.J., Wong-Leung J., Yong C.-K., Docherty C.J., Paiman S., Gao Q., Tan H.H., Jagadish C., Lloyd-Hughes J., Herz L.M. (2012). Ultralow surface recombination velocity in InP nanowires probed by terahertz spectroscopy. Nano Lett..

[37] Zekry A. (1998). Electronic Devices.

[38] Sze S.M., Li Y., Ng K.K. (2021). Physics of Semiconductor Devices.

[39] Streetman B.G., Banerjee S. (2000). Solid State Electronic Devices.

[40] Neamen D.A. (2003). Semiconductor Physics and Devices: Basic Principles.

[41] Kittel C., McEuen P. (2018). Introduction to Solid State Physics.

[42] Fang H., Hu W. (2017). Photogating in low dimensional photodetectors. Adv. Sci..

[43] Zhang A.Y. (2010). Silicon Nanowire Phototransistor: Designing, Fabricating and Characterizing a High Responsivity, Broadband Photodetector. Ph.D. Thesis.

[44] Boylestad R.L., Nashelsky L. (2012). Electronic Devices and Circuit Theory.

[45] Atlas User’s Manual.

[46] McGregor D.S., Hermon H. (1997). Room-temperature compound semiconductor radiation detectors. Nuclear Instruments and Methods in Physics Research Section A: Accelerators, Spectrometers. Detect. Assoc. Equip..

[47] McMorrow D., Als-Nielsen J. (2011). Elements of Modern X-ray Physics.

[48] Persson K.M., Berg M., Borg M.B., Jun W., Johansson S., Svensson J., Jansson K., Lind E., Wernersson L.E. (2013). Extrinsic and Intrinsic Performance of Vertical InAs Nanowire MOSFETs on Si Substrates. Mater. J..

[49] Barnaby H.J., Schrimpf R.D., Galloway K.F., Li X., Yang J., Liu C. (2016). Displacement damage in bipolar junction transistors: Beyond Messenger-Spratt. IEEE Trans. Nucl. Sci..

[50] Liu Y. (2014). Radiation damage in InP/InAs heterostructure nanowires. Appl. Phys. Lett..

[51] Ding F. (2014). X-ray irradiation induced defects and the effect on the photoluminescence of InP nanowires. Nanoscale Res. Lett..

[52] Verschuuren M.A. (2012). Radiation-induced surface states in InP nanowires. Nano Lett..

[53] Bhattacharya I. (2017). Nanophotonic Devices Based on Indium Phosphide Nanopillars Grown Directly on Silicon. Ph.D. Thesis.

